# *Faecalibacterium duncaniae* A2-165 regulates the expression of butyrate synthesis, ferrous iron uptake, and stress-response genes based on acetate consumption

**DOI:** 10.1038/s41598-023-51059-3

**Published:** 2024-01-10

**Authors:** Sophie Verstraeten, Séverine Layec, Sandrine Auger, Catherine Juste, Céline Henry, Sawiya Charif, Yan Jaszczyszyn, Harry Sokol, Laurent Beney, Philippe Langella, Muriel Thomas, Eugénie Huillet

**Affiliations:** 1 Micalis Institute, INRAe, AgroParisTech, Université Paris-Saclay, Jouy-en-Josas, France; 2 Paris Center for Microbiome Medecine (PaCeMM) FHU, AP-HP, Paris, France; 3 Institute for Integrative Biology of the Cell (I2BC), CEA, CNRS, Université Paris-Saclay, Gif-Sur-Yvette, France; 4 UMR PAM, INRAe, Université Bourgogne Franche-Conté, AgroSup Dijon, Dijon, France

**Keywords:** Bacteria, Transcriptomics, Biomarkers

## Abstract

The promising next-generation probiotic *Faecalibacterium prausnitzii* is one of the most abundant acetate-consuming, butyrate-producing bacteria in the healthy human gut. Yet, little is known about how acetate availability affects this bacterium’s gene expression strategies. Here, we investigated the effect of acetate on temporal changes in the transcriptome of *F. duncaniae* A2-165 cultures using RNA sequencing. We compared gene expression patterns between two growth phases (early stationary vs. late exponential) and two acetate levels (low: 3 mM vs. high: 23 mM). Only in low-acetate conditions, a general stress response was activated. In high-acetate conditions, there was greater expression of genes related to butyrate synthesis and to the importation of B vitamins and iron. Specifically, expression was strongly activated in the case of the *feoAABC* operon, which encodes a FeoB ferrous iron transporter, but not in the case of the *feoAB* gene, which encodes a second putative FeoAB transporter. Moreover, excess ferrous iron repressed *feoB* expression but not *feoAB*. Lastly, FeoB but not FeoAB peptides from strain A2-165 were found in abundance in a healthy human fecal metaproteome. In conclusion, we characterized two early-stationary transcriptomes based on acetate consumption and this work highlights the regulation of *feoB* expression in *F. duncaniae* A2-165.

## Introduction

Bacteria in the genus *Faecalibacterium* (phylum *Bacillota*^1^, class *Clostridia,* family *Oscillospiraceae*) are commensal, strictly anaerobic bacteria, that are ubiquitous and abundant in the gastrointestinal tracts of humans and animals^2,3^. This genus is currently recognized as one of the most important commensals for human health^4,5^. According to the latest phylogenetic analysis^6^, the genus *Faecalibacterium* is composed of six species, of which *prausnitzii* is the best known for its effects on human health. The most extensively studied strain of *F. prausnitzii*, A2-165, has been recently reclassified and placed into a new species, *duncaniae* sp. nov.^6^. Interest in *Faecalibacterium* has increased over the last decade, mostly in response to the pioneering study of Sokol et al*.* reporting a depletion of *F. duncaniae* A2-165 in Crohn’s disease patients^7^. Since this report, many studies have confirmed reduced abundances of *Faecalibacterium* in patients with inflammatory bowel disease and metabolic diseases^8,9^, which has led to consider members of this genus into candidates for the development of diagnostic, prognostic, preventive or therapeutic approaches^10^.

In the healthy human colon, *Faecalibacterium* are among the main bacteria responsible for the consumption of acetate and the production of butyrate. Butyrate is the preferred energy source for colonocytes and has anti-inflammatory properties that are generally considered to be beneficial to intestinal health^11,12^. Recently, we demonstrated that *dact3*, an host gene linked to the Wnt/JNK pathway, mediates these *F. prausnitzii* anti-inflammatory effects^13^. In addition, several peptides originating from a single 15-kDa protein (Microbial Anti-inflammatory Molecule, MAM) have been identified in the supernatant of *F. duncaniae* A2-165 cultures^14–16^, and these have been shown to alleviate chemically induced colitis in mice^17,18^. The key enzyme for butyrate production is butyryl-*CoA*:acetate *CoA*-transferase. It generates butyrate and acetyl-*CoA* from extracellular acetate and intracellular butyryl-*CoA*^2,19–21^. In addition to generating butyrate, this process also promotes growth, thus explaining the growth-stimulating effects of culture supplementation with a high amount of acetate (33–50 mM)^2,21,22^. In co-culture models, acetate cross-feeding between *Faecalibacterium* and acetate-producing bacteria such as *Bifidobacteria adolescentis*^23^ or *Blautia hydrogenotrophica*^21^ has been observed to enhance butyrate formation. A positive correlation between acetate consumption and butyrate production was also established in vivo between *F. duncaniae* A2-165 and *Bacteroides thetaiotaomicron*^24^.

In the human gut, acetate is the most abundant short-chain fatty acid largely generated by the bacterial fermentation of dietary fibers. Its concentration fluctuates depending on various parameters, including gut transit time, diet, age, health status, and microbiota composition^25–27^. To cope with such changes*, Faecalibacterium* have presumably developed regulatory systems that sense and respond to acetate levels. These adaptive strategies would necessarily involve a well-coordinated gene expression network that takes into account both the physiological state of the bacteria (latency, exponential, stationary phases) and the environmental conditions (availability of acetate and other nutrients, physicochemical signals such as oxygen tension, pH, temperature). Although several recent studies have investigated the transcriptome of *F. duncaniae*^21,28,29^, none has examined the effects of acetate across growth phases. In light of its proposed vital role in intestinal health, this information is critical for understanding the metabolism of *Faecalibacterium* and how this might affect human health.

Here, we investigated the effect of acetate on temporal changes in the transcriptome of *F. duncaniae* A2-165 cultures using RNA sequencing. Next, we characterized the regulation of two *feoB* genes—which encode well-known ferrous iron transporter—in response to the availability of acetate and ferrous sulfate. Finally, using an novel integrated approach, we searched for and identified FeoB peptides specific to the A2-165 strain in the healthy human fecal metaproteome. The general workflow of our study can be found as Supplementary Fig. S5 online.

## Results

### Experimental design for acetate-dependent transcriptome analysis

**Figure 1:**
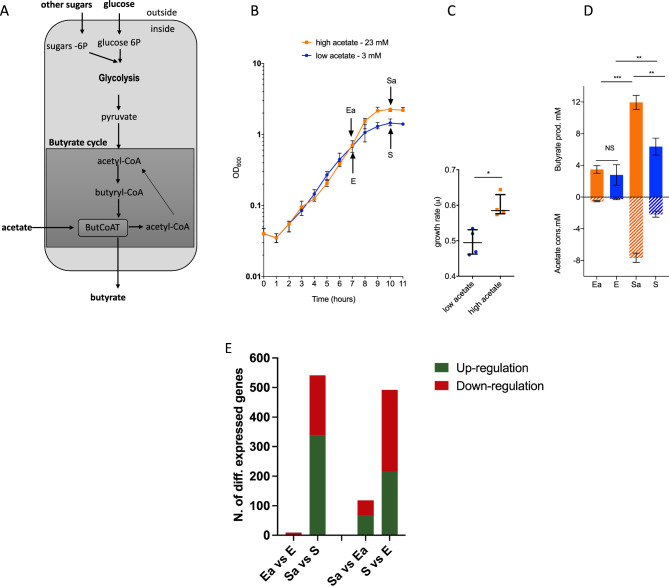
Acetate growth effect and butyrate production in *F. duncaniae* A2-165 cultures. (**A**) Schematic view of the importance of acetate in the butyrate biosynthetic pathway. The production of butyrate is dependent on glycolysis and the butyrate cycle. The key enzyme for butyrate production is butyryl-*CoA*:acetate *CoA*-transferase (ButCoAT), which consumes extracellular acetate to produce butyrate and acetyl-CoA from butyryl-CoA. (**B**) Growth kinetics of *F. duncaniae* A2-165 under high- and low-acetate conditions. Growth kinetics are shown for bacteria cultured in BHIS medium with 23 mM acetate (high-acetate, orange color) or with 3 mM acetate (low-acetate, blue color). Arrows indicate time points of sampling for the RNA-Seq transcriptome analysis. Sampling was performed after 7 h (late exponential phase, E and Ea for low- and high-acetate conditions, respectively) and 10 h (early stationary phase, S and Sa for low- and high-acetate conditions, respectively) of growth. N = 4, median with interquartile range (IQR) is shown. (**C**) Individual growth-rate measures in high/low-acetate conditions. N = 4, raw data, median with IQR are shown. (**D**) SCFA concentrations (expressed as molarity) of E/Ea and S/Sa culture supernatants. Butyrate production is presented as positive values while acetate consumption is presented as negative values. Acetate values were normalized with the acetate value of BHIS medium, which contains 3 mM of acetate. N = 4, median with IQR is shown. (**E**) Overview of RNA-Seq data. The number of (differentially expressed) DE genes for each comparison is presented in the form of a bar chart. Two-group comparisons were conducted between high- and low-acetate conditions in the late exponential phase (Ea vs. E) and early stationary phase (Sa vs. S). Additionally, two-group comparisons were performed between the early stationary and late exponential phases in high-acetate (Sa vs. Ea) and low-acetate (S vs. E) conditions. log_2_FC ≥ |2|, FDR-adjusted p-value ≤ 0.01.

Acetate consumption is the main driver of butyrate production by genus *Faecalibacterium*, as shown in Fig. 1A. To gain insight into the effect of acetate on the transcriptome of *F. duncaniae* A2-165, two sets of cultures were established in media that contained low (3 mM) or high (23 mM) concentrations of acetate (see “Methods” section). The resulting growth kinetics are shown in Fig. 1B. In both acetate conditions, there was no difference in growth kinetics over the first 7 h, and the stationary growth phase was reached after 9 h of culture in both treatments. In the early stationary phase, the biomass in high-acetate conditions (Sa) was about 1.5-fold higher than in low-acetate conditions (S) (2.30 ± 0.13 OD_600_ vs. 1.50 ± 0.18 OD_600_, N = 4, *p* < 0.05). Moreover, the growth rate was 1.25-fold higher in high-acetate conditions (Fig. 1C), suggesting that the general metabolism of *F. duncaniae* A2-165 was highly active in these cultures. Indeed, the generation time was reduced by about 25 min in this group compared to that of low-acetate cultures (1.67 h vs. 2.08 h). In high- and low-acetate conditions, we quantified butyrate production and acetate consumption in both the late exponential growth phase (Ea and E, respectively) and the early stationary growth phase (Sa and S, respectively) (Fig. 1D). In both acetate conditions, butyrate production was significantly higher in the early stationary phase than in the late exponential phase. Moreover, in the late exponential phase (7 h of growth) there was no difference between acetate conditions in either butyrate production or acetate consumption. In contrast, in the early stationary phase (10 h of growth), butyrate production and acetate consumption were significantly different between acetate conditions (butyrate: 11.94 mM ± 0.89 for Sa, 6.37 mM ± 1.07 for S, *p* < 0.01). Moreover, after 10 h of growth, almost all acetate had been consumed in the low-acetate cultures (− 2.16 mM ± 0.38), whereas only one-third of the acetate had been consumed in the high-acetate cultures (− 7.66 mM ± 0.58). The acetate limitation experienced in the low-acetate conditions strongly affected the growth of *F. duncaniae* A2-165 (Fig. 1B,C). We thus investigated the acetate-growth effect at the transcriptional level in both acetate-limited and acetate-saturated conditions using RNA-Seq. For this, gene expression profiles were analyzed at both E/Ea and S/Sa time points (Fig. 1B).

### Overview of transcriptomes in low- and high-acetate conditions

The RNA-Seq datasets contained a total of 2882 genes out of the 3013 predicted in the RefSeq database (i.e., over 95%). Of these, 1159 genes were found to be differentially expressed (DE) (log_2_ fold change (FC) ≥ |2| and FDR-adjusted p-value ≤ 0.01) between conditions. All DE genes are presented in Supplementary Tables S1–S4 online. Eight of these genes were differentially expressed in early exponential cultures, compared to 541 in the late stationary phase (339 and 202 transcripts up- and down-regulated, respectively, Fig. 1E), which is consistent with the different patterns of growth kinetics. This result clearly demonstrates that, under our experimental conditions, acetate concentration (3 vs. 23 mM) had little impact on transcriptomic profiles in *F. duncaniae* A2-165 for the first 7 h of growth (i.e., up to the late exponential growth phase), but had a significant impact when the cultures entered the stationary phase of growth. This prompted us to perform a detailed analysis of the adaptive changes that occur in the transcriptome in response to acetate availability between 7 and 10 h of growth. As shown in Fig. 1E, there was a large difference in the adaptive response between high-acetate (Sa/Ea, 118 DE genes) and low-acetate (S/E, 492 DE genes) conditions in this three-hour period. Specifically, we found that, under high-acetate conditions, only 67 and 51 genes were up- and downregulated, respectively, whereas under low-acetate conditions, 216 and 276 genes were up- and downregulated, respectively. When comparing the DE transcript lists in low- and high-acetate conditions (see Supplementary Fig. S6 online), it clearly appeared that the acetate-limiting condition triggered a larger adaptive transcriptional response at the onset of the stationary growth phase compared to the acetate-saturated conditions, in our growth model.

**Figure 2:**
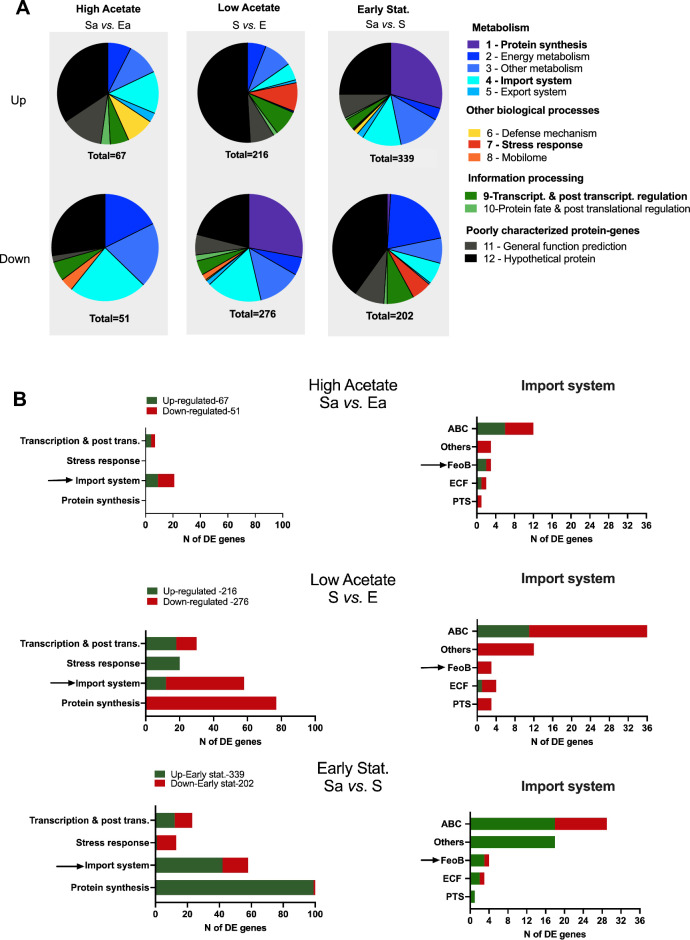
Transcriptional responses of *F. duncaniae* A2-165 to acetate. (**A**) Pie charts of up- and downregulated genes showing the relative frequency (%) of each functional category. The number of genes in each category is shown in Table S5 in Supplementary file 2 and the total number of DE genes is shown below each pie-chart. The right part of the panel shows the representative color for each category. The category “Other biological processes” contained the metabolism subcategories “Amino acid”, “Amino sugar”, “Nucleotide”, “Vitamin and Cofactor”, “Lipid”, and “Cell wall”, as well as “Bacterial cell division processes”. The “Energy metabolism” category corresponds to the conversion of carbon sources and acetate to ATP and the carbon skeleton and includes glycolysis and the butyrate pathway. See Tables S2–S4 in Supplementary file 2 for data on DE genes (gene ID, COG number, protein annotation, log_2_FC, padj value, category). Bold characters indicate category selection in this study. (**B**) Description of relevant functional categories and import system subclassification in high, low and early stationary acetate transcriptomes. ABC (ATP-binding cassette) transport family. Others: other transport system family, FeoB (Ferrous) iron transport system family. PTS: phosphoenol pyruvate transferase system family, ECF (Energy Coupling Factor) transport family. Arrows indicate “Import system” category selection and FeoB transport system selection in this study.

Using the COG and PATRIC databases, we classified DE genes into 12 functional categories (see Supplementary Table S5 online). As shown in Fig. 2A, for all transcriptomes (high-acetate, low-acetate, and early stationary), the largest category (27.2–58.8% of genes) was the superclass “Poorly characterized protein-genes”, which included the categories “Hypothetical proteins” and “General function prediction”. It is likely that these unknown DE genes contribute to the adaptive responses of *F. duncaniae* A2-165 as cells enter the stationary phase. In addition, clear differences between low- and high-acetate transcriptomes were observed with respect to the categories “Protein synthesis”, “Energy metabolism”, “Import system”, “Defense system”, “Stress response”, and “Transcription and Post-transcriptional regulation” (Fig. 2A). Interestingly, in acetate-limiting conditions (S vs. E), the highest proportions of up- and downregulated genes were related to “Stress response” (9.3%, i.e., 20 DE genes) and “Protein synthesis” (27.9%, i.e., 77 DE genes) respectively, whereas in acetate-saturated conditions (Sa vs. Ea), the highest proportions of affected genes were related to “Import system” (13.4%, i.e., 9 upregulated genes, and 23.5%, i.e., 12 downregulated genes).

Overall, our transcriptome analysis strongly suggests that *F. duncaniae* A2-165 is able to tightly regulate the expression of metabolic and stress-response genes according to acetate levels when cells enter the stationary phase, in our growth conditions. Remarkably, in all comparative transcriptional analyses, the CG447_03795 gene encoding the MAM protein did not appear among the DE genes; the transcription of this gene was quite high and unchanged between acetate conditions as well as between the growth phases examined here (see Supplementary Fig. S7 online).

We analyzed in detail the categories with the highest gene count belonging to the TOP 3, in low- and high- acetate transcriptomes (see Table S5 in Supplementary Information). These categories include ‘Protein synthesis’ and ‘Stress response’ in low acetate transcriptomes, while they encompass ‘Import system’ in high acetate transcriptomes. Differentially expressed transcriptional regulators may yield clues as to how the adaptive response is regulated. We concentrated on the ‘Transcription and Post-transcriptional Regulation’ category in low acetate growth conditions to associate putative transcriptional regulators with potential target genes.

### A general stress response under acetate-limiting conditions

As depicted in Fig. 2B, we clearly observed significant differences in the transcriptional responses of *F. duncaniae* A2-165 under acetate-limiting condition compared with acetate-saturated condition. Interestingly, in low-acetate condition, we found a high number of downregulated transcripts in the “Protein synthesis” category (77 DE genes, log_2_FC range: 2–5, Table S3) that were not detected in high-acetate condition. These were mainly transcripts involved in translation machinery, such as translation initiation/elongation factors and aminoacyl-tRNA synthetases/transferases, but also included genes involved in ribosome biogenesis and/or stability. These findings likely indicated a major slowdown of translational processes under low-acetate growth conditions as *F. duncaniae* cells entered the stationary phase.

We also noted a drastic shift in the “Stress response” category (20 DE genes, Table S3) which again was not observed in high-acetate condition. Seven of these genes (log_2_FC range: 2.2–2.9) encoded chaperone proteins involved in protein remodeling, in the repair of proteins following stress damage, or even in the ubiquitin machinery and proteasome. The remaining 13 (log_2_FC range: 2.1–6.2) genes were associated with type II toxin/antitoxin (TA) systems^30^. More specifically, we observed the upregulation of four putative operons for RelE TA systems, one putative operon for a TA module in the Doc family, two genes encoding antitoxin proteins, and one gene encoding a toxin protein. Of these, the DinJ/YafQ system (CG447_14090/14095) was the most activated TA system (log_2_FC: 6.1–6.2). Overall, our data suggest that cells of *F. duncaniae* A2-165 in low-acetate condition experienced severe general stress upon entry to the early stationary growth phase.

Bacteria employ different transcriptional regulators and sigma transcription factors to respond to changing environments. In the low-acetate transcriptome examined here, we found a higher number of upregulated genes encoding transcriptional regulators and sigma transcription factors (18 DE genes, log_2_FC range: 2.1–5.5, see Supplementary Table S3 online) compared to high-acetate condition (4 DE genes, log_2_FC range: 3.0–5.0, see Supplementary Table S4 online). Moreover, in all bacteria studied thus far, the global regulator that mediates the general stress response is a specialized sigma factor^31^. In the low-acetate transcriptome, we specifically found five upregulated genes encoding sigma factors (CG447_02225/05485/06440/06875/08160, see Supplementary Table S3 online), while in high-acetate conditions there was only one, which was also upregulated in low-acetate conditions (CG447_02215, Tables S4-S5). It is thus possible that these five sigma factors could be involved in the general stress response described above when cells enter the stationary phase.

### Deciphering import systems under high- and low-acetate conditions

Transcription of transporter genes is usually regulated in response to substrate availability^32^. As shown in Fig. 2B, we clearly observed two distinct import system transcriptomes of *F. duncaniae* A2-165, under acetate-limiting and saturated conditions. These findings likely indicated two major adaptation responses to substrate availability, according to acetate conditions. A large number of genes (46) belonging to the “Import system” category were found to be downregulated under low-acetate conditions (see Supplementary Table S3 online); this number was four-fold higher than under high-acetate conditions (see Supplementary Table S4 online). These were mainly transporter genes in the ABC superfamily (25 DE genes), in the sodium-dependent transporter family (5 DE genes), in the ECF subfamily (3 DE genes) and in the FeoB family (4 DE genes) (Supplementary Table S3 online, Fig. 2B). Within the ECF group, we noticed a reduction in gene expression related to B vitamins uptake (*bioY* gene, CG447_11745, involved in biotin import, log_2_ FC − 4.14; *ribU* gene, CG447_14300, log_2_ FC − 3.92, involved in riboflavine import). We noticed a considerable reduction in gene expression related to ferrous iron uptake (*feoA*_1_*A*_2_*BC* putative operon, CG447_12740-55, log_2_ FC range: − 4.9 to 5.9), and ferric iron uptake (*fhu* putative operon, CG447_03300-10, log_2_FC range: − 6.1 to 8.0). This suggested that the expression of major systems of iron transport was severely impaired in low-acetate conditions. Conversely, we found that expression of the *feoA*_*1*_*A*_*2*_*BC* operon was upregulated under high-acetate condition (log_2_FC: 1.5–2.1, Fig. 2B). This was particularly marked in early stationary transcriptome, in which we observed an even higher fold-change in expression (log_2_FC: 4.5–5.9, Sa vs*.* S, Supplementary Table S2 online, Fig. 2B). Many studies have explored the role of the FeoB high affinity transporters in ferrous iron uptake in iron-poor environments^33,34^. Our result suggests that in the early stationary phase in an acetate-saturated environment, ferrous iron is no longer abundant, and activation of the *feoAABC* system may allow *F. duncaniae* A2-165 cells to maintain iron homeostasis in these conditions.

### Under high-acetate conditions, *feoB* is the most transcribed *feo* homolog

**Figure 3:**
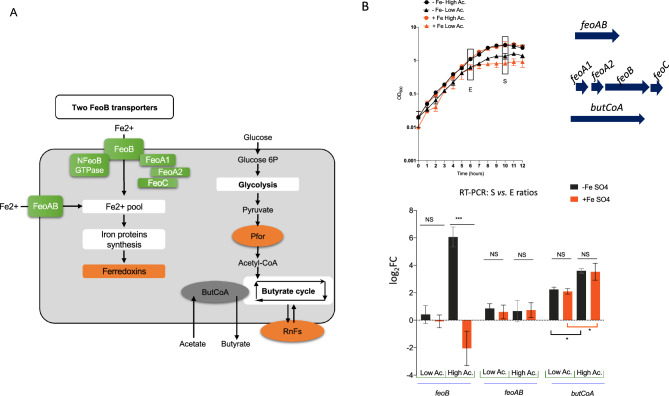
Expression analysis of *feoB*, *feoAB* and *butCoA* by RT-PCR. (**A**) Schematic representation of ferrous iron uptake, iron protein synthesis, glycolysis, and butyrate metabolism pathway. The FeoB membrane transporter, NFeoB domain with GTPase activity, and FeoA1, FeoA2, and FeoC cytosolic proteins are indicated. In the FeoB systems that have been described to date, cytosolic proteins are required for ferrous iron acquisition but the exact mechanism is not yet known^33^. The glycolysis pathway and *Faecalibacterium*’s genus-specific butyrate production pathway (shown as butyrate cycle) are shown. Pyruvate is converted to acetyl-CoA by pyruvate:ferredoxin oxidoreductase (PFOR) and the energy generated during butyrate production is conserved through the buildup of a proton gradient via the activity of a membrane-associated NADH:ferredoxin oxidoreductase encoded by two putative *rnf* operons (*rnfDEA*, CG447_09840-830, *rnfCDGEAB* CG447_10905-930)^37^. Ferredoxin proteins (orange color). See Tables S2–S4 in Supplementary file 2 for data on DE genes (gene ID, COG numbers, protein annotation, log_2_FC, padj value, category). (**B**) Effect of the addition of ferrous sulfate on the transcription of *feoB, feoAB*, and *butCoA* genes, quantified using RT-PCR. Growth kinetics of *F. duncaniae* strain A2-165 in the presence or absence of ferrous sulfate (50 µM) in high-acetate (23 mM acetate) and low-acetate (3 mM) growth conditions. Square: culture sampling at T6 (exponential (E) growth phase) and T10 (early stationary (S) growth phase) time points. Genetic organization of the putative *feoA*_*1*_*A*_*2*_*BC* operon is shown.

In order to confirm our hypothesis of limited iron availability in the high-acetate culture conditions, we analyzed *feoB* expression in the presence of excess ferrous sulfate (Fig. 3). Figure 3A illustrates ferrous iron uptake and iron homeostasis in *F. duncaniae*. As a control, we also analyzed gene expression in low-acetate conditions, as well as the expression of *feoAB* (CG447_08795, log_2_FC: − 4.3, see Supplementary Table S4 online) and *butCoA*, which encodes the terminal enzyme required for butyrate production (Fig. 3A). A new set of *F. duncaniae* A2-165 cultures was prepared with high or low levels of acetate and with or without ferrous sulfate, and sampling was performed as previously described (i.e., in the late exponential phase, indicated by E, and the early stationary phase, indicated by S, Fig. 3B). Figure 3B presents the results regarding the expression ratios (S vs. E) of *feoB*, *feoAB*, and *butCoA* obtained under the different conditions. These clearly demonstrated that the *feoB* expression was significantly triggered (log_2_FC: 6.0) during the early stationary phase, only in the high-acetate condition, and in the absence of ferrous sulfate supplementation. Moreover, its expression was totally repressed in the presence of ferrous sulfate (log_2_FC: − 2.0). Overall, the *feoB* regulation was dependent on the availability of an iron source in the medium, with strong activation and repression in the absence and presence of ferrous sulfate, respectively. For the *feoAB* gene, the expression ratios were similar in the absence or presence of an iron source in both acetate growth conditions, indicating that the regulation of *feoAB* is independent of iron availability in our growth condition. For the *butCoA* gene, we observed a positive growth phase effect in both acetate and iron conditions with stronger activation in the high-acetate conditions (Fig. 3B). Overall, these results demonstrate that regulation of the expression of *feoAABC* and *feoAB* differs significantly and suggest that the FeoB and FeoAB systems of *F.* *duncaniae* A2-165 play nonredundant roles in ferrous iron acquisition.

### FeoB peptides from strain A2-165 are found in abundance in a healthy human fecal metaproteome

**Figure 4:**
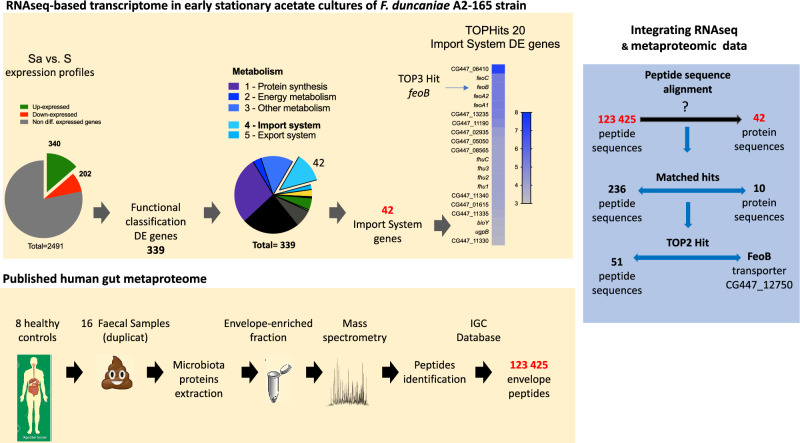
Workflow for the search for FeoB peptides in the healthy human gut metaproteome. RNA-Seq transcriptome of early stationary acetate cultures of *F. duncaniae* strain A2-165. (**A**) The general workflow employed for RNA-Seq in the current study: from identification of DE genes in the early stationary phase (Sa vs S comparison) to *feoAABC* operon selection. (**B**) Published human gut metaproteome. The general workflow employed in a published study of the gut metaproteome. The data were obtained from eight healthy subjects included in a study of intestinal bowel diseases^35^. (**C**) Integrating RNA-Seq and metaproteomic data. The general workflow employed to integrate RNA-Seq and metaproteomic data in the current study and the presentation of data for *F. duncaniae* A2-165.

We were interested in studying *feoB* expression in the human gut microbiome. For this, we analyzed a fecal metaproteomic dataset obtained from eight healthy individuals, which included 123,425 peptides from the envelope fraction of the gut microbiota (Fig. 4)^35^. The metaproteomics/transcriptomics data can be found in Supplementary Tables S6–S8 online. Among these peptides, 236 matched with 10 of the 42 transporter genes that we had found to be upregulated in high-acetate conditions. Remarkably, the second highest degree of protein coverage (43.6%, 51 peptides) was found for the FeoB transporter. Of those 51 peptides, 9 were specific to a single protein in the metaproteomic dataset ‘a5.b59.a1’ (see Supplementary Table S7-2 ‘CG447_12750 FeoB’ and Supplementary Fig. S8 online), which was identified as FeoB of the A2-165 strain (K04759 in the KEGG orthology database). In addition, among the 573 identified proteins from A2-165 strain, FeoB belongs to the top 20 hits in term of spectra number (see Supplementary Table S8 online). On the other hand, FeoAB protein exhibited a fivefold lower number of spectra corresponding to the 284th position in the A2-165 proteins list (see Supplementary Table S8 online). Overall, this demonstrates that FeoB, but not FeoAB, from A2-165 is found in abundance in this healthy human fecal metaproteome^35^.

## Discussion

### Two early stationary lifestyles of *F. duncaniae* A2-165

Here, we investigated the adjustments in the bacterial transcriptome when cells of *F. duncaniae* A2-165 entered the stationary growth phase in low- and high-acetate conditions. To our knowledge, this is the first physiological study based on transcriptomic and metabolic analyses that focuses on the entry into the stationary growth phase of *F. duncaniae* and, moreover, of a bacterium belonging to the *Faecalibacterium* genus.

Given that *Faecalibacterium* is a strictly anaerobic bacterium, cultivation presents challenges^36^. We have limited options for monitoring the growth of *F. duncaniae*, which include a semi-defined medium (“Yeast, Casitone, Fatty acid” named YCFA medium of Duncan laboratory^2^) with slow growth and low biomass, or rapid growth with high biomass in the rich complex BHIS medium. We opted for the latter method to enable accurate and precise tracking of stationary phase entry over a 12–14 h period.

Comparative analysis of the high- and low-acetate transcriptomes showed two different metabolic responses regarding the number of genes and their functions. Given that acetate is a major growth factor (i.e. major energy source)^37^, this seems consistent. Because of the lack of relevant data from the literature with which to compare our results, we chose to focus on the strongest and clearest effects, i.e., high fold-changes in expression. In the future, it will be necessary to study (i) the functions carried by the weaker adjustments in the transcriptome (i.e. lower fold change), (ii) this adaptive response in continuous culturing using chemostats, (iii) this adaptive response in co-culture models with acetate-producing bacteria.

Our experimental design enabled the identification of a general stress response under low acetate growth conditions. A characteristic of the exponential-stationary transition is the induction of stress proteins adapted for general stress condition. The general stress response has been very well characterized in model bacteria such as *Escherichia coli* and *Bacillus subtilis*^38^, but this protective adaptive response is not known in *Faecalibacterium*. Notably, a general stress response was observed only in low-acetate conditions; concomitantly with the general stress response, a negative regulation of protein synthesis genes and a positive regulation of genes encoding type II toxin/antitoxin modules were also observed. As the majority of type II toxins inhibit translation^30^, we can hypothesize that the former observation may be the consequence of the latter. Sigma B is the sigma factor involved in the control of the general stress response^38^ in many bacteria including model bacteria and several anaerobic bacteria such as *Clostridioides difficile*^39^. Remarkably, the gene encoding Sigma B is not present in A2-165 genome. Our functional analysis led to a list of genes encoding Sigma factors, that could be involved in the control of this protective response in low-acetate conditions. To our knowledge, this study represents the first description of a general stress response, in the early stationary phase, in acetate-limiting conditions in *Faecalibacterium*.

### FeoB transporters search strategy

In the second part of our study, we chose to study bacterial transporters of the Feo family because the transport function of ferrous iron and its regulation are well characterized^32,33^. For this, we focused on the fecal metaproteome of healthy individuals, previously published^35^. This metaproteome was of interest in our study of the *F. duncaniae* A2-165 import systems because it targeted bacterial envelope proteins. We combined high-resolution mass spectrometry of fecal specimens with RNA-seq of *F. duncaniae* A2-165 pure culture. Our metaproteomic analysis shows a significant difference in abundance between FeoB and FeoAB, suggesting variations in the regulation of *feoAABC* and *feoAB* expression in the context of an healthy gut microbiome. In conclusion, our search strategy allows us to capture the major A2-165 Feo import systems, namely the FeoB transporter. In the future, this search strategy can be extended to investigate Feo transporters in other bacteria of the genus *Faecalibacterium* using the same metaproteomic dataset.

### Iron homeostasis and butyrate production

In many environments, including the human gut, iron is a limiting nutrient for growth, and high-affinity uptake systems play a central role in ferrous iron homeostasis^40^. Maintenance of ferrous iron homeostasis is crucial for the production of butyrate^37^ because this process requires two iron-binding ferredoxins as illustrated in Fig. 3A. Here, we demonstrated that *butCoA* expression was strongly upregulated regardless the supplementation of cultures with ferrous sulfate. This result suggests that, in the early stationary growth phase in high-acetate conditions, mechanisms of ferrous iron homeostasis were well established in *F. duncaniae* A2-165.

Interestingly, together with upregulation of *feoAABC*, we also detected upregulation of a flavodoxin-encoding gene (see Supplementary Table S4 online). This is consistent with previous studies reporting that iron-free flavodoxin replaces iron-sulfur ferredoxin under iron-limited conditions^40,41^. Because flavodoxin and ferredoxin both mediate electron transfer in redox processes, the use of flavodoxin could free up iron for utilization by other iron-dependent enzymes. In other word, flavodoxins are key players in maintenance of ferrous iron homeostasis in iron-limited environment^40,41^. For example, in *C. difficile*, the production of flavodoxin is tied to iron homeostasis in iron-limited conditions^42^. In addition, the switch to flavodoxin as electron transfer protein may have significant effects on the activity of numerous enzymes that use flavodoxin as a redox partner. For example, in *B. subtilis*, flavodoxin is the redox partner for the acyl lipid desaturase involved in fatty-acid desaturation^43^. To date, similar data are not available for *Faecalibacterium*, but the impact of flavodoxins on the activity of certain metabolic pathways, including butyrate pathway, as well as the link with iron availability, merits further investigation.

## Methods

### Strain used for experimental culture

This study used a stock of *Faecalibacterium duncaniae* A2-165 (DSM No. 17677, DSMZ collection, Braunschweig, Germany) originally isolated from human fecal stool by S. H. Duncan (University of Aberdeen, United Kingdom)^2^ and maintained by V. Robert, MICALIS Institute, INRAE, France.

### Cultivation experiments of *F. duncaniae*

*F. duncaniae* A2-165 was grown under anaerobic conditions (anaerobic chamber: N_2_ = 90%, CO_2_ = 5%, and H_2_ = 5%) at 37 °C in BHIS medium (also named LYBHI in our previous studies), which is brain heart infusion broth (BHI, 37 g/L, Difco) supplemented with yeast extract (5 g/L, Difco), cellobiose (1 mg/mL, Sigma), maltose (1 mg/mL, Sigma), and cysteine (0.5 mg/mL, Sigma)^17^. The strain was thawed and grown from a 10% inoculation in 10 mL BHIS for 24 h at 37 °C, followed by a 1% inoculation in 10 mL BHIS for overnight growth (16–18 h). The experimental cultures were then created using 2% inoculation in 50 mL BHIS medium with (BHISAc) or without the addition of sodium-acetate solution (20 mM, Sigma^2,37^) at the time of inoculation. Growth kinetics were monitored by measuring the OD_600_ nm every hour. The low acetate condition (3 mM) corresponded to the basal amount of acetate that we measured in the BHIS growth medium. The high acetate condition (20 mM added to the medium, i.e. final concentration 23 mM), was based on the previous in vitro studies of Duncan et al.^2^ and Heinken et al.^37^, as well as our own measurements of acetate concentration in human feces (median value 20 mM, see Table 1 of Martin et al.^27^).

### Addition of ferrous sulfate to *F. duncaniae* culture

A fresh solution of ferrous sulfate (FeSO_4_-7H_2_O, Sigma) was prepared with deionized water and filter-sterilized. The experimental cultures were created using 2% inoculations in 50 mL BHIS or BHISAc media. Ferrous sulfate (50 µM, Sigma) was added 3 h after inoculation.

### Short-chain fatty acid (SCFA) production and consumption

Gas chromatography (7890B, Agilent Technologies) was used to identify the short-chain fatty acids present in culture supernatants. The SCFAs of interest in this study were acetate and butyrate. An internal standard of 2-ethylbutyrate was used to normalize the data in each run. Measurements of SCFA concentrations in the bacterial samples were normalized using the basal SCFA levels of BHISAc and BHIS media from the same experiment.

### Collection of RNA samples

For both growth conditions, cells and culture supernatant were sampled in the late exponential growth phase (7 h after inoculation) and early stationary growth phase (10 h after inoculation) for RNA extraction (cells) and SCFA analysis (supernatant). At the times of sampling, dilutions were created of all cultures and plated on BHIS agar for quantification of colony-forming units (CFUs).

### RNA extraction

The single-step RNA isolation method was used, with TRIzol reagent and the FastPrep-24™ 5G instrument. For RNA-Seq and RT-PCR analyses, 2 mL of each culture was harvested and centrifuged (30 s, 13,000 rpm) in the Freter chamber, and cell pellets were snap-frozen in liquid nitrogen. 1 mL of TRIzol reagent (phenol-based RNA extraction buffer, Ambion, ThermoFisher Scientific) and 0.4 g of glass beads (0.1 mm, Bühler, ThermoFisher Scientific) were added to the defrosted pellet, and cell lysis was performed with a FastPrep-24™ 5G instrument (MP Biomedicals) (speed 6, 40 s). RNA precipitation, washing, and solubilization was carried out following the TRIzol protocol. Residual DNA from the RNA preparations was enzymatically removed using TURBO DNA-free (Ambion, United Kingdom). RNA quality was assessed with the Agilent 6000 Nano kit using an Agilent 2100 bioanalyzer (Stratagene, Agilent Technologies, France); RIN values were in the 7.7–8.5 range. Total RNA was purified from three biological replicates for RNA-Seq experiments and for RT-qPCR analysis. Extracted RNA samples were stored in RNAase/DNAse-free water (Ambion, United Kingdom) at − 80 °C.

### Library construction and Illumina sequencing

Total RNA quality was assessed using an Agilent Bioanalyzer 2100 and the RNA 6000 Pico kit (Agilent Technologies). Directional RNA-Seq libraries were constructed using the TruSeq Stranded Total RNA library prep kit, with bacterial Ribo-Zero reagents (Illumina), following the manufacturer’s instructions; 500 ng of total RNA were used (I2BC HTS platform, CNRS, Gif-sur-Yvettes, France). After the Ribo-Zero step, the samples were checked on the Agilent Bioanalyzer to verify rRNA depletion. The quality of the libraries was assessed on an Agilent Bioanalyzer 2100, using an Agilent High Sensitivity DNA Kit. Libraries were pooled in equimolar proportions and sequenced in a paired-end 2 × 75-pb run on an Illumina NextSeq500 instrument (I2BC HTS platform, CNRS, Gif-sur-Yvettes, France). Demultiplexing was performed with bcl2fastq2 v2.18.12. Adapters were trimmed with Cutadapt v1.15, and only reads longer than 10 pb were kept for further analysis.

### Mapping and identification of differentially expressed genes

Reads were mapped on the genome of *F. duncaniae* strain A2-165 (GenBank: CP022479.1) with BWA 0.6.2-r126, and were counted using the subread feature of Counts v1.5.2. Analyses of differential expression were performed in R using DESeq2 (I2BC HTS platform, CNRS, Gif-sur-Yvettes, France). In total, 2900 genes—including 2819 protein-coding sequences, 63 tRNA genes, and 18 rRNA genes, were mapped. Only the first two groups were analyzed in this study, so that a total of 2882 genes were considered. These were further filtered to retain only ORFs that showed a four-fold change in expression (absolute value Log_2_FC > = 2) (up/down) between treatment conditions. RNA-Seq datasets can be found on the database Omics Dataverse under the DOI accession number BDNCT2 (10.15454/BDNCT2).

### RT-PCR analysis

We generated cDNA from 1 μg of total RNA using the High Capacity cDNA Reverse Transcription kit (Fisher Scientific, France) and random hexamers. The quality of cDNA was checked with an Agilent 6000 Pico kit and an Agilent 2100 Bioanalyzer (Stratagene, Agilent Technologies, France). We carried out qPCR in duplicate, in a reaction volume of 20 μL containing 500 pg of cDNA, 15 μL of SYBR^®^ Green PCR Master Mix (Applied Biosystems, Courtaboeuf, France), and 300 nM of each gene-specific primer. The primers were designed with Primer Express^®^ (version 3.0). We generated standard curves for each set of primers, using serial dilutions (four dilutions) of cDNA obtained from a mix of total RNA collected in BHISAc and BHIS. Amplification was carried out with an ABI^®^ PRISM 7900 thermal cycler (Applied Biosystems), with the following thermal profile: 2 min at 50 °C, 10 min at 95 °C, and 40 cycles of 15 s at 95 °C and 60 s at 60 °C. The specificity of each PCR amplicon was checked by melting curve analysis. Here, the *feoB* (CG447_12750, F5′-tgatcttcaacctgctgtgc, R5′-gccacgatggtgaagaagtt), *feoAB* (CG447_08795, F5′-ataccgaagtcaccgacctg R5′-ctccatcagctgcatcgtta) and *butCoA* (CG447_01820, F5′-actttgttctgggcgcatac, R5′-ggtcagtcccttcaggttca) primers used in this study. The *efp* gene (CG447_05125), encoding the translation elongation factor P, was used for normalization with the primers *efpF* (5-gttgagttccagcacgtgaa-3) and *efpR* (5-aaagcctgagggaactttgc-3). The relative change in gene expression was recorded as the ratio of normalized target concentrations and was calculated with the comparative ΔΔCt method^44^. The mean values for three independent experiments are presented.

### Protein sequence annotations, functional classification

Protein annotation data from the PATRIC database^45^ (PATRIC genome ID 853.173) were used for functional classification. As the functional subsystem coverage in PATRIC was low (29%), EggNOG database was used for COG-based functional classifications using the COG system (https://www.ncbi.nlm.nih.gov/research/COG).

We created a simplified classification scheme with 12 categories; the largest metabolism classes (protein synthesis, energy, and import system) were retained while smaller metabolism classes (cell wall metabolism, lipid metabolism, amino acid metabolism, amino sugar metabolism, nucleotide metabolism, vitamin and cofactor metabolism) were merged into an “Other metabolism” category. The “Import system” and “Export system” classes encompassed all membrane transport systems involved in the uptake/export processes of nutrients, micronutrients, ions, and unknown compounds. “Stress response” category encompassed toxin/antitoxin systems and chaperones. The TC-BLAST tool (TCD database https://www.tcdb.org/) was used for the determination of the number of TMS (putative TransMembrane Segment) and for the import system family assignment. To characterize gene similarity, homology, and gene context, NCBI BLAST was used, in particular for analysis of *feo* genes.

### Fecal metaproteomics analysis

We conducted an analysis of 16 published available metaproteomes derived from the envelope fractions of fecal microbiota obtained from eight healthy donors without any family history of gastrointestinal disease and no medication use^35^. The details of the metaproteomic method has been described previously^35^. To note, a minimum of two distinct peptides identified across all samples in the dataset was set to validate a protein in order to exclude proteins of weak proof of presence.

### Combined A2-165 transcriptomics and fecal metaproteomics analysis

Each of the 123,425 peptide sequences identified in the fecal metaproteomes was matched against each of the 42 query protein sequences identified in this RNA-seq analysis (see Supplementary Tables S6–S8 online) in the R environment.

### Statistical analysis

Normally distributed data are presented as mean ± standard deviation (SD), skewed data as median with interquartile range (IQR), and categorical data as frequencies with proportions. *T*-tests for independent samples and one-way analysis of variance (ANOVA) (or Kruskal–Wallis when not normally distributed) were performed for continuous variables. Data were represented and analyzed with GraphPad Prism 8.

### Supplementary Information

Supplementary Information.

Supplementary Tables S1–S5.

Supplementary Tables S6–S8.

## Data Availability

The data that support the findings of this study are openly available in database Omics Dataverse at 10.15454/BDNCT2 [DOI accession number BDNCT2]. All of the material is owned by the authors and/or no permissions are required.
