# Supplementary material for: Faecalibacterium duncaniae A2-165 regulates the expression of butyrate synthesis, ferrous iron uptake, and stress-response genes based on acetate consumption
Source: Sci Rep. 2024 Jan 10;14:987. doi: 10.1038/s41598-023-51059-3 (PMC10781979; doi:10.1038/s41598-023-51059-3)
Supplement: Supplementary file 1 — Supplementary Information. [file 41598_2023_51059_MOESM1_ESM.pdf]

Faecalibacterium duncaniae A2-165 regulates the expression of butyrate synthesis, ferrous iron uptake, and stress-response genes based on acetate consumption

Sophie Verstraeten[1], Séverine Layec[1], Sandrine Auger[1,2], Catherine Juste[1], Céline Henry[1], Sawiya Charif[1], Yan Jaszczyszyn[3], Harry Sokol[1,2], Laurent Beney[4], Philippe Langella[1,2], Muriel Thomas[1,2], Eugénie Huillet[1,2]*

[1]*Micalis Institute, INRAe, AgroParisTech, Université Paris-Saclay, Jouy-en-Josas, France*
[2]*Paris Center for Microbiome Medecine (PaCeMM) FHU, AP-HP, Paris, France*
[3]*Institute for Integrative Biology of the Cell (I2BC), CEA, CNRS, Université Paris-Saclay, Gif-sur-Yvette, France.*
[4]*UMR PAM, INRAe, Université Bourgogne Franche-Conté, AgroSup Dijon, Dijon, France*
*Corresponding author: Eugenie Huillet, eugenie.huillet@inrae.fr

Supplementary information

1. Supplementary Figures S5-S8
2. Legends for Supplementary Tables S1-S5
3. Legends for Supplementary Tables S6-S8

Figure S5. Scheme summarizing the experimental set-up and methods used in the study: in light grey, the first part of the study; in dark grey, the second part of the study.

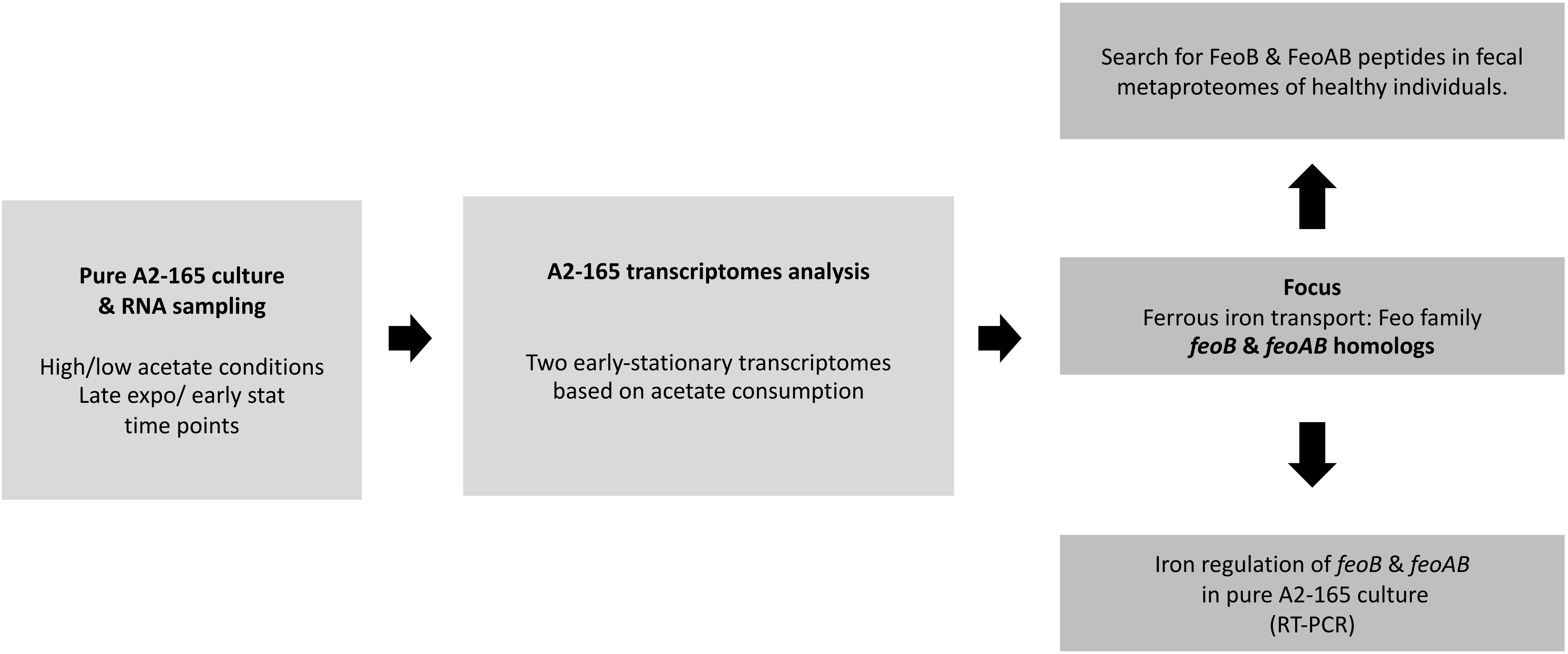

Figure S6

Legend, Figure S6.

Venn diagrams of upregulated and downregulated genes under two conditions. Each diagram was created based on two lists of DE genes. These lists contained 67, and 216 DE genes for the upregulated diagram (left part) and 51, and 276 DE genes for the downregulated diagram (right part). Of the 283 upregulated genes (Sa *vs.* Ea and S *vs.* E lists), 21 genes were held in common, while 46 and 195 genes were specific to the high- and low-acetate conditions, respectively. Of the 327 downregulated genes (Sa *vs.* Ea and S *vs.* E lists), 26 genes were held in common, while 25 and 250 genes were specific to the high- and low-acetate conditions, respectively. $\log_2 FC \geq |2|$, FDR-adjusted p-value ≤ 0.01. Differentially expressed (DE)

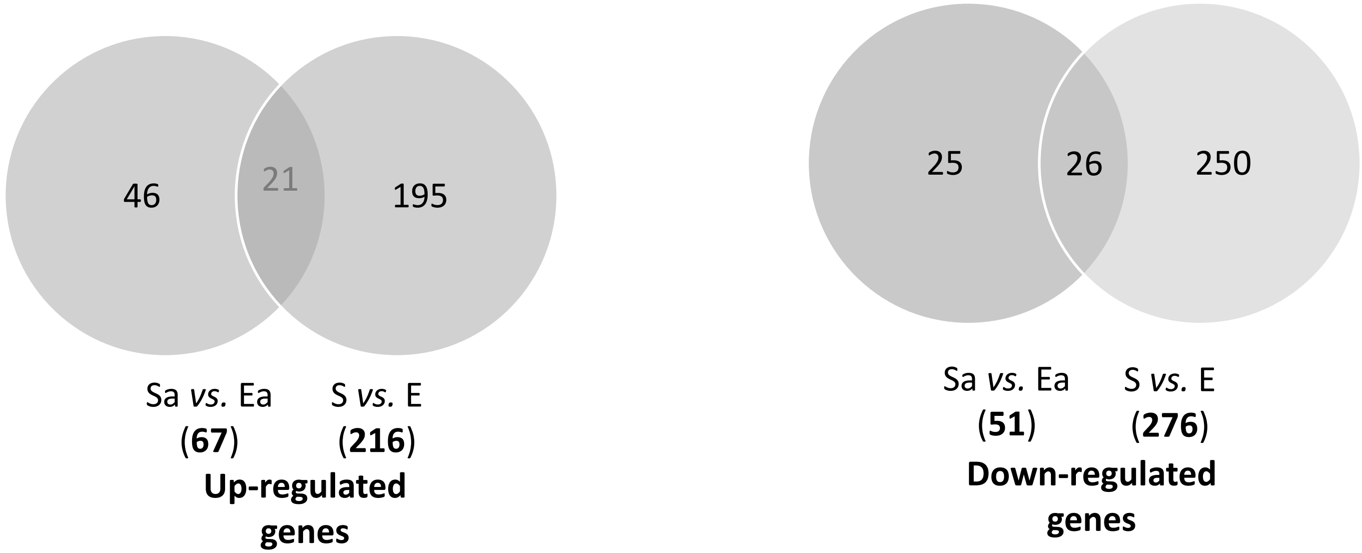

Figure S7. Expression of the CG447_03795 gene, encoding the MAM protein, across the late exponential (E/Ea time points) and early stationary (S/Sa time points) phases in low- and high- acetate conditions, using RNA-Seq.

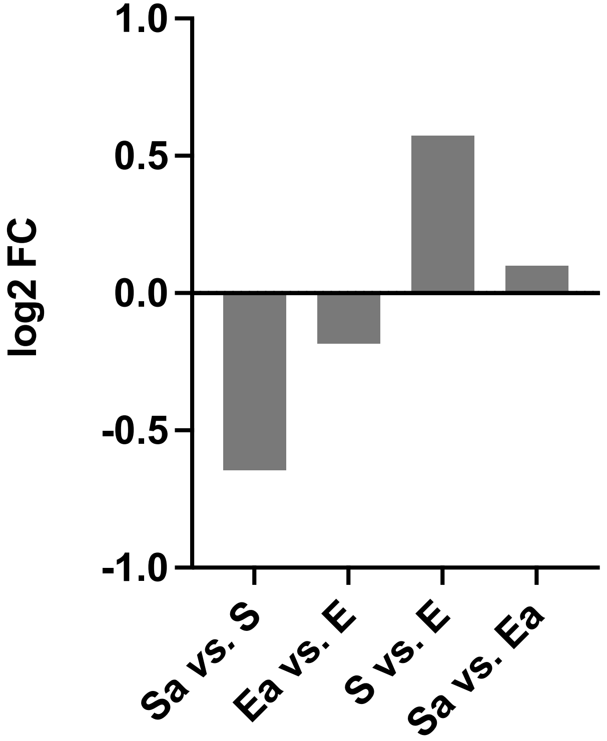

Figure S8. Localization of the detected peptides (underlined &bold) that match with the FeoB sequence (729 aa) of *F. duncaniae* strain A2-165 and that match with other FeoB sequences of *Faecalibacterium* bacteria and gut commensal bacteria (IGC2 database; see Supplementary Table S7) .

>fig|853.173.peg.2751|CG447_12750| Ferrous iron transporter FeoB

MSIKIALAGNPNCGKTTLFNNLTGSNQYVGNWPGVTVEKKEGKLKGDKDVIIQDLPGIYSLSPYT
LEEVVSRTYLVKEKPDAILNIIDGTNIERNLYLTTQLIELGIPVVMAVNMIDLVRKNGDKIDLK**K**
LSSELGCQAVEISALKGEGTEAAAKAAVAAAQKQKAGELPHVFTGSVEHAIAHIEESIQGKVDDR
FLRWYAVKLFER**DEKVVEELKLDKALADHIDEHIKDCEKEMDDDAESIITNQRYSYINGVVDK**AV
KKK**ARVEHLTASDKIDQIVTNR**VLALPIFALVMFLMYSLSMGTSIADGGWAIGTFATDWTNDVLF
GEIVPGALGGFLESIGVAGWLYGLIMDGIVAGVGAVLGFVPQMLVLFFLLSILEDVGYMSRVAFI
MDRIFRRFGLSGKSFIPVLVGTGCGVPGVMASRTIENERDRRMTIMTTCFIPCGAKMPIIGLIAG
AMFGGSSLVAVSAYFIGMAAIICSGVILKKTKLFAGDPAPFVMELPAYHVPAWGNVFRATWERGW
SFIKRAGSVILAATVVLWFLQGFGFENGAFGMVEDQDNSVLAAIATKIAWIFAPLGFGNWRATVA
SVSGLIAKENVVGTFGVLYHFGGELSENGDEIWAAVAQDYTALSAYAFMIFNLLCAPCFAAMGAI
KREMNNGKWTAIAIGYMCALAYCAALVVYQIGGLITGEVGFNFFTIVAIVIFAAFLYLMFRPNKY
VGDNEVKIDVSKIK

Legends for Supplementary Tables S1-S5

RNA-Seq-based transcriptome analyses

Legends common to Supplementary Tables S1-S4:

- Growth kinetics of *F. duncaniae* A2-165 was performed in BHIS medium with 23 mM acetate (high-acetate) or with 3 mM acetate (low-acetate). RNA Sampling was performed after 7 hours (late exponential phase, E and Ea for low- and high-acetate conditions, respectively) and 10 hours (early stationary phase, S and Sa for low- and high-acetate conditions, respectively) of growth.

- DE: differentially expressed
- log2 FC is ≥ |2|, FDR-adjusted p-value ≤ 0.01.
- For some genes the log2FC are between 1.5 and 2 when these genes belong to the same functional locus
- Columns from left to right contain RefSeq ID, gene name if known, COG number, PATRIC functional annotation, the expression fold change in log2, the adjusted p-value and the functional category.

Legends

- **Supplementary Table S1. Description of the transcriptome under Late Exponential conditions: list of 8 *F. duncaniae* A2-165 DE genes.**

Two-group comparison, Ea *vs.* E : 1 up-and 7 down -regulated genes.

- **Supplementary Table S2. Description of the transcriptome under Early Stationary conditions: list of 541 *F. duncaniae* A2-165 DE genes.**

Two-group comparison, Sa *vs.* S : 339 up and 202 down -regulated genes.

- **Supplementary Table S3. Description of the transcriptome under Low Acetate conditions: list of 492 *F. duncaniae* A2-165 DE genes.**

Two-group comparison S *vs.* E: 216 up and 276 down -regulated genes.

- **Supplementary Table S4. Description of the transcriptome under High Acetate conditions: list of 118 *F. duncaniae* A2-165 DE genes.**

Two-group comparison, Sa *vs*. Ea: 67 up and 51 down -regulated genes.

- **Supplementary Table S5**. **Distribution of functional categories**.

Legends for Supplementary Tables S6-S8

Combined transcriptomics and metaproteomics analysis

Overview: We analyzed a fecal metaproteomic dataset obtained from eight healthy individuals, which included 123,425 peptides from the envelope fraction of the gut microbiota, previously published in Henry et *al*, 2022, DOI 10.3390/cells11081340**.**
Each of the 123,425 peptide sequences identified in the human metaproteomes was matched against each of the 42 protein sequences identified in this RNA-seq analysis (Sa *vs.* S comparison, Early Stationary transcriptome, Supplementary Table S6) in the R environment.

Supplementary Table S6. List of 42 *F. duncaniae* A2-165 up-regulated genes (query sequences) encoding putative import system proteins in high acetate conditions in early stationary growth phase (comparison Sa vs. S).
Columns from left to right contain RefSeq ID (in yellow, matched hit), gene name if known, PATRIC functional annotation, the expression fold change in log2, the adjusted p-value, the functional category, the simplified subclassification, the TCD subclassification and the number of putativeTransMembrane Segment (TMS). The TC-BLAST tool was used for the determination of the number of TMSs and for the import system family assignment. TCD database https://www.tcdb.org/

Supplementary Table S7. List of the 236 hit peptides found in the metaproteome of healthy individuals that match 10 *Faecalibacterium* transport proteins using IGC2 database.
Legend:
-The corresponding peptide, protein and accession identifiers in IGC2 are indicated in columns B to D, separated by a coma if more than one. The corresponding taxonomy in IGC2 is indicated in columns O and P, separated by ';;;' if more than one.
-Specific peptides (found in a unique protein of the IGC2 database), are colorized in deep red. By filtering those specific peptides, we can predict the taxonomy of the transporters (see sheets 2:6 of the table; note that transporter CG447_11340 has no specific peptide).

Supplementary Table S7-1. Specific peptides of CG447_12410 protein
Supplementary Table S7-2. Specific peptides of CG447_12750 protein (FeoB protein, see **Supplementary Fig. S4)**
Supplementary Table S7-3. Specific peptides of CG447_11330 protein
Supplementary Table S7-4. Specific peptides of CG447_12415 protein
Supplementary Table S7-5. Specific peptides of CG447_08360 protein

Supplementary Table S8: list of 573 *F. duncaniae* A2-165 proteins and metaproteins found in the metaproteome of healthy individuals (Henry *et al*, 2022, DOI 10.3390/cells11081340)

Legend: Proteins were identified using *F. duncaniae* A2-165 UniProt database**.**
Columns from left to right contain: Sub-group ID, group of proteins sharing common peptides; Accession Fd A2-165 , accession number of the *F. duncaniae* A2-165 identified protein using UniProt database; Description, functional description of the identified protein; Coverage : percentage of the sequence coverage; Size, number of amino acids of the protein; Specific Spectra : number of unique distinct spectra allowing the identification of the protein, Specific sequences: number of unique distinct peptide sequences only assigned to this protein within the group.

- In green, FeoB: sub-group, b17.a1; accession ID UniProt, tr|C7H443|C7H443_FAEPA; accession ID PATRIC, CG447_12750, 729 A.A., 464 spectra, 57 sequences.

- in yellow, FeoAB: sub-group, c284.a1; accession ID UniProt, tr|C7H1S4|C7H1S4_FAEPA accession ID PATRIC CG447_08795, 780 A.A.), 11 spectra, 6 sequences.
